# Hepatic G Protein-Coupled Receptor 180 Deficiency Ameliorates High Fat Diet-Induced Lipid Accumulation via the Gi-PKA-SREBP Pathway

**DOI:** 10.3390/nu15081838

**Published:** 2023-04-11

**Authors:** Yunhua Zhang, Ziming Zhu, Lijun Sun, Wenzhen Yin, Yuan Liang, Hong Chen, Yanghui Bi, Wenbo Zhai, Yue Yin, Weizhen Zhang

**Affiliations:** 1 Department of Physiology and Pathophysiology, School of Basic Medical Sciences, and Key Laboratory of Molecular Cardiovascular Science, Ministry of Education, Peking University, Beijing 100191, China; zyh@bjmu.edu.cn (Y.Z.); zzming@pku.edu.cn (Z.Z.);; 2 The Key Laboratory of Xinjiang Endemic & Ethnic Diseases and Department of Biochemistry, Shihezi University School of Medicine, Shihezi 832002, China; 3 Department of Pharmacology, School of Basic Medical Sciences, and Key Laboratory of Molecular Cardiovascular Science, Ministry of Education, Peking University, Beijing 100191, China

**Keywords:** GPR180, nonalcoholic fatty liver disease, SREBP1, SREBP2

## Abstract

Single-nucleotide polymorphisms in G protein-coupled receptor 180 (GPR180) are associated with hypertriglyceridemia. The aim of this study was to determine whether hepatic GPR180 impacts lipid metabolism. Hepatic GPR180 was knocked down using two approaches: *Gpr180*-specific short hairpin (sh)RNA carried by adeno-associated virus 9 (AAV9) and *alb-Gpr180*^−/−^ transgene established by crossbreeding *albumin-Cre* mice with *Gpr180^flox/flox^* animals, in which Gpr180 was specifically knocked down in hepatocytes. Adiposity, hepatic lipid contents, and proteins related to lipid metabolism were analyzed. The effects of GPR180 on triglyceride and cholesterol synthesis were further verified by knocking down or overexpressing *Gpr180* in Hepa1-6 cells. *Gpr180* mRNA was upregulated in the liver of HFD-induced obese mice. Deficiency of *Gpr180* decreased triglyceride and cholesterol contents in the liver and plasma, ameliorated hepatic lipid deposition in HFD-induced obese mice, increased energy metabolism, and reduced adiposity. These alterations were associated with downregulation of transcription factors SREBP1 and SREBP2, and their target acetyl-CoA carboxylase. In Hepa1-6 cells, *Gpr180* knockdown decreased intracellular triglyceride and cholesterol contents, whereas its overexpression increased their levels. Overexpression of *Gpr180* significantly reduced the PKA-mediated phosphorylation of substrates and consequent CREB activity. Hence, GPR180 might represent a novel drug target for intervention of adiposity and liver steatosis.

## 1. Introduction

As a center of lipid synthesis, expenditure, and circulation, the liver is critical for lipid homeostasis. Under physiological conditions, the balance between lipid synthesis, β-oxidation, and export is precisely controlled by a series of pathways ranging from hormones and G protein-coupled receptors (GPCRs) to nuclear receptors and transcription factors. Dysfunction of several GPCRs, and associated heterotrimeric G proteins, increases lipid droplet accumulation in hepatocytes, leading to subsequent development of liver steatosis. For example, global GPR55 deficiency increases adiposity, stimulates hepatic lipogenesis, and promotes the development of liver steatosis [1]. Moreover, global deletion of Gα12 promotes fat accumulation in the liver of mice. Similarly, the steatotic liver of patients with nonalcoholic fatty liver disease (NAFLD) have decreased levels of Gα12 expression [2]. However, whether orphan GPCRs contribute to the development of liver steatosis remains largely unknown.

GPR180 is an orphan GPCR that is also known as the intimal-thickness-related receptor (ITR). This receptor was originally found to promote the proliferation of vascular smooth muscle cells, leading to subsequent vascular intimal thickening [3]. As such, it is considered a risk factor for atherosclerosis [4]. In addition to vascular remodeling, GPR180 reportedly plays an important role in tumorigenesis [5,6,7,8,9,10] and pupil development [11]. Recent genome-wide association studies (GWASs) also indicate that single nucleotide polymorphisms (SNPs) in GPR180 are related to hypertriglyceridemia [12,13]. Expression quantitative trait locus (eQTL) analysis of 1183 liver samples has further revealed that GPR180 alleles rs2298058 and rs9561643 are associated with a significant increase in *Gpr180* expression and could be relevant to elevated triglyceride levels [14]. Collectively, these observations suggest that hepatic GPR180 may alter lipid metabolism.

This study aimed to investigate whether hepatic GPR180 contributes to the control of lipid metabolism and its underlying mechanism. Our results demonstrate that deficiency of GPR180 in the mouse liver significantly decreases adiposity and liver steatosis induced by a high-fat diet. Furthermore, knockdown of *Gpr180* in Hepa1-6 cells inhibits lipid synthesis. The effect of hepatic GPR180 on lipid metabolism is likely mediated by sterol regulatory element-binding proteins (SREBPs), including SREBP1 and SREBP2, in a PKA-dependent manner. The findings of this study suggest that GPR180 could be a potential novel drug target for the treatment of adiposity and liver steatosis.

## 2. Methods

### 2.1. Viruses and Small Hairpin (sh)RNAs

Adeno-associated viruses of serotype 9 (AAV9) carrying GPR180-specific shRNA (AAV9-shRNA GPR180) or control-scrambled shRNA (AAV9-shRNA scramble) were packaged by Weizhen Biotechnology Co., Ltd. (Jinan, China). To improve the knockdown efficiency, the following four GPR180-specific shRNA sequences: (1) 5′-CTCCCAAATTCAAGATGCTGTA-3′, (2) 5′-TGCTTCAGCCTTAGCTAATTA-3′, (3) 5′-GCTTCAGCCTTAGCTAATTAC-3′, and (4) 5′-GCTCTTGCTGATTGTCTTACG-3′, were simultaneously inserted into the pAV4-in-1-GFP vector. 

### 2.2. Laboratory Animals

All experimental procedures were performed in accordance with the regulations on laboratory animal management of the Ministry of Health of the People’s Republic of China (Document No. 55, 2001). Male C57BL/6J mice (6 weeks old) were purchased from the Laboratory Animal Department of the Peking University Health Science Center. Mice were maintained under standard conditions with a 12 h light–dark cycle (light 06:00–18:00, darkness 18:00–06:00) and temperature of 21–25 °C. After one week of acclimatization, mice were randomly divided into four groups: fed normal control diet (NCD; Beijing Keao Xieli Feed Co., Ltd., Beijing, China) and injected with AAV9-shRNA GPR180 or AAV9-shRNA scramble at a dose of 5 × 10^11^ vg/mouse via the tail vein, or fed high-fat diet (HFD; Research Diet, D12492, 60% kcal from fat, 5.24 kcal/g) and injected with AAV9-shRNA GPR180 or AAV9-shRNA scramble. Body weight and food intake were measured weekly. Before performing the oral glucose tolerance test (OGTT) and insulin tolerance test (ITT), mice were fasted for the time indicated; however, they were allowed free access to water. Mice were sacrificed after 12.5 weeks as described previously [15].

The transgenic mice *Albumin-cre* and *Gpr180^flox/flox^* were purchased from Shanghai Model Organisms Center (Shanghai, China). *Albumin-Gpr180*^−/−^ (*Gpr180*^−/−^) mice and *Gpr180^flox/fox^* littermates were generated via homozygous crossing. The husbandry conditions of transgenic mice were consistent with those of mice treated with adeno-associated virus. Six-week-old male transgenic mice were then assigned to receive HFD for 16 weeks.

The liver tissues of diabetic mice (db/db) and nondiabetic mice (db/m) were prepared as described previously [16].

### 2.3. Measurement of Energy Metabolism and Fat Contents

Mice were subjected to energy metabolism analysis using a metabolic cage (Harvard Apparatus, Holliston, MA, USA) and body fat content analysis with a magnetic resonance imaging (MRI) system 3.0T (Siemens, Munich, Germany) at the Peking University Health Science Center.

### 2.4. Analysis of Triglyceride and Cholesterol Contents

Triglyceride and cholesterol contents in plasma, tissues, and cells were determined using a triglyceride assay (#A110-2-1) and total cholesterol assay (#A111-2-1) kits (Nanjing Jiancheng Bioengineering Institute, Nanjing, China), according to the manufacturer’s instructions.

### 2.5. Oil Red O Staining

Mice were sacrificed by intraperitoneal injection of 1% pentobarbital sodium (100 mg/kg), followed by bilateral thoracotomy. The liver was washed with phosphate-buffered solution, then fixed with 4% paraformaldehyde, embedded with OCT compound, and sectioned at 5 μm. The frozen sections were warmed at room temperature for 20 min, washed with double-distilled (dd) H_2_O for 1 min, rinsed with 60% isopropanol for 20 s, and stained with oil red O working solution in the dark for 60 min. Sections were washed with ddH_2_O for 3 min, mounted with 90% glycerol, and stored at 4 °C until microscopy analysis.

### 2.6. RT-PCR

Total RNA was extracted from mouse tissues or cells using an RNA extraction kit (Applygen, Beijing, China) and reverse-transcribed into cDNA using a reverse transcription kit (Yeasen, Shanghai, China). The expression levels of specific genes were analyzed by quantitative RT-PCR using a quantitative fluorescence kit (Vazyme, Nanjing, China) and primers specific for *Actb* (forward 5′-CAGCCTTCCTTCTTGGGTAT-3′ and reverse 5′-TGGCATAGAGGTCTTTACGG-3′) and *Gpr180* (forward 5′-TGTCAGAATCAACAACGTAGCAG-3′ and reverse 5′-TTGTCGGCATAGAACACTCGC-3′).

### 2.7. Western Blotting

Western blotting was performed as previously described [17]. Primary antibodies against Laminb1(#12987-1-AP) and β-actin (#66009-1-Ig) were obtained from Proteintech (Wuhan, China), while those against SREBP1 (#A15586), SREBP2 (#A13049), HMGCS1 (#A3916), ACC (#A15606), and FASN (#A0461) were purchased from Abclonal (Wuhan, China). Phospho-PKA substrates (RRXS/T; #9624) was purchased from Cell Signaling Technology (Danvers, MA, USA). Secondary fluorescent IRDye 800CW antibodies were obtained from Bioss Corporation (Beijing, China). ImageJ software (v. 1.8.0_112, NIH, Bethesda, MD, USA) was used to quantify protein expression.

### 2.8. Cell Cultures and Treatment

Hepa1-6 murine hepatoma cells (ATCC#CRL-1830) were cultured in high-glucose Dulbecco’s Modified Eagle’s Medium (Invitrogen, Carlsbad, CA, USA) supplemented with 10% fetal bovine serum (Hyclone, Logan, UT, USA), streptomycin sulfate (100 mg/mL), and penicillin (100 units/mL) at 37 °C and 5% CO_2_. To investigate the function of GPR180 in lipid metabolism, cells were transfected with pcDNA-GFP, pcDNA-*Gpr180* (Ruibo Technology Co., Ltd., Beijing, China), *Gpr180* small interfering siRNA (GCTCAGCCTTAGCTAATTAC; Suzhou Hongxun Biotechnology Co., Ltd., Suzhou, China), or control siRNA, respectively, and treated with a mixture of oleic acid (100μM) and palmitic acid (50 μM). Cells were harvested after 24 h and analyzed for mRNA and protein expression, as well as lipid contents.

For the protein kinase A (PKA) activity assay, Hepa1-6 cells were transfected with the *Gpr180* overexpression plasmid for 30 h, cultured in serum-free medium for 12 h, and then in 10% serum-containing medium for the time indicated. Cells were then analyzed for measurement of activity at different time points by PKA substrate antibody immunoblotting. 

### 2.9. Dual-Luciferase Reporter Assay

The resuspended HEK-293T cells (ATCC#CRL-3216) were seeded into a 24-well plate (2 × 10^5^ cells/well), subsequently transfected with 300 ng pGL3-CREB (CREB promoter-luciferase reporter), 30 ng pRL-CMV, and 170 ng pcDNA-*Gpr180* or pcDNA3.1 plasmids (provided by Ruibo Technology) with lipo8000 (Beyotime, Shanghai, China). Luciferase assays were performed after a 24 h transfection using the Dual Luciferase Reporter Assay System (E1910, Promega (Madison, WI, USA)). The CREB-driven promoter luciferase activity was determined by normalizing the activity of firefly luciferase against Renilla luciferase.

### 2.10. Statistical Analysis

The experimental results were expressed as mean ± standard error of the mean (SEM). Graphs were constructed, and statistical analyses performed using the GraphPad Prism5 software (La Jolla, CA, USA). Differences between the groups were analyzed by one-way ANOVA and Student’s *t*-test. *p* < 0.05 denotes for statistical significance.

## 3. Results

### 3.1. Gpr180 Is Upregulated in the Livers of Obese Mice

**Figure 1:**
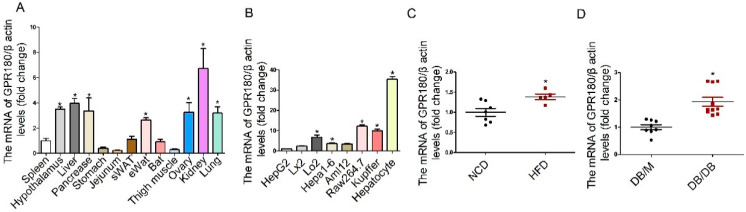
GPR180 expression profile in mouse tissues and liver cells. (**A**) GPR180 expression in different mouse tissues and (**B**) cell lines; presented as fold-change compared with that in the spleen or HepG2 cells, respectively. (**C**) GPR180 expression in the livers of mice fed NCD or HFD, as well as (**D**) diabetic (db/db) and nondiabetic (db/m) mice. Results are presented as the mean ± SEM and analyzed by one-way ANOVA and unpaired Student’s *t*-test; *n* = 5–10, * *p* < 0.05.

Large-scale meta-analysis of GWAS identified the *Gpr180* loci associated with plasma triglyceride levels in population (Table 1). To determine whether GPR180 participates in lipid metabolism, we first analyzed its mRNA expression in multiple mouse tissues. *Gpr180* was abundant in the kidney, liver, hypothalamus, pancreas, lungs, and ovaries (Figure 1A). In liver, it was expressed in primary hepatocytes and macrophages (Raw264.7 and Kupffer cells). Relative to primary hepatocytes, human and mouse hepatocyte cell lines, as well as stellate cell line Lx2, expressed significantly lower levels of *Gpr180* (Figure 1B).

Next, we evaluated *Gpr180* transcription in the livers of obese and normal mice. Levels of *Gpr180* mRNA were significantly upregulated in the livers of HFD-induced obese mice relative to lean mice fed NCD (Figure 1C). Further, its expression was markedly increased in the liver of db/db diabetic mice compared with control (db/m) animals (Figure 1D). These results indicate that the *Gpr180* gene is expressed in the liver, particularly within hepatocytes, and its level is upregulated in obese mice.

### 3.2. Hepatic Gpr180 Deficiency Alleviates HFD-Induced Obesity

**Figure 2:**
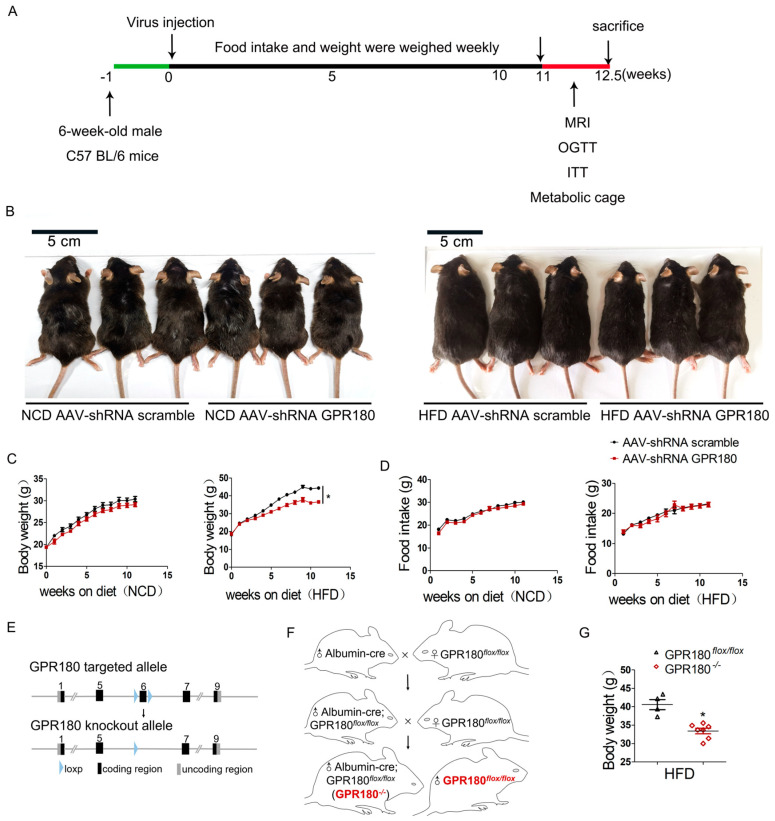
Effects of hepatic *Gpr180* knockdown on body weight and food intake in mice fed NCD or HFD. (**A**) Experimental design of in vivo experiments. (**B**) Body size in mice fed NCD or HFD (*n* = 8–10) treated with AAV9-*shGpr180* or control scramble shRNA. (**C**) Body weight (**D**) and food intake. (**E**,**F**) Strategy to generate *GPR180*^−/−^ transgenic mice. (**G**) Body weight in GPR180*^flox/flox^* and GPR180^−/−^ mice fed HFD (*n* = 4–7). Results are shown as mean ± SEM and analyzed by one-way ANOVA and Student’s unpaired *t*-test; * *p* < 0.05.

To knockdown hepatic *Gpr180*, mice fed NCD or HFD were administered AAV9-shRNA *Gpr180* via tail vein injection. AAV9-shRNA scramble was used as the control. Body weight and food intake were monitored weekly for a period of 12.5 weeks (Figure 2A). Hepatic *Gpr180* deficiency caused a significant reduction in the body sizes and weights in mice fed HFD (Figure 2B,C), whereas no effect was observed on food intake (Figure 2D). These effects were not detected in mice fed NCD (Figure 2B–D). Meanwhile, glucose metabolism in mice fed NCD or HFD was evaluated using oral glucose tolerance test (OGTT) and insulin tolerance test (ITT). Hepatic *Gpr180* deficiency enhanced insulin sensitivity in mice fed HFD (Supplementary Figure S1).

To generate transgenic mice with hepatocyte-specific knockout of *Gpr180*, the loxp site was inserted at both ends of exon 6 of the *Gpr180* gene (Figure 2E). *Gpr180^flox/flox^* mice were crossbred with *Albumin-cre* transgene to obtain *Albumin-Gpr180*^−/−^ (*Gpr180*^−/−^) mice (Figure 2F). After 16 weeks of HFD feeding, the body weights of *Gpr180*^−/−^ mice were significantly lower than those of *Gpr180^flox/flox^* animals (Figure 2G). These results suggest that hepatic *Gpr180* deficiency alleviates HFD-induced obesity.

### 3.3. Hepatic Gpr180 Knockdown Enhances Energy Expenditure

**Figure 3:**
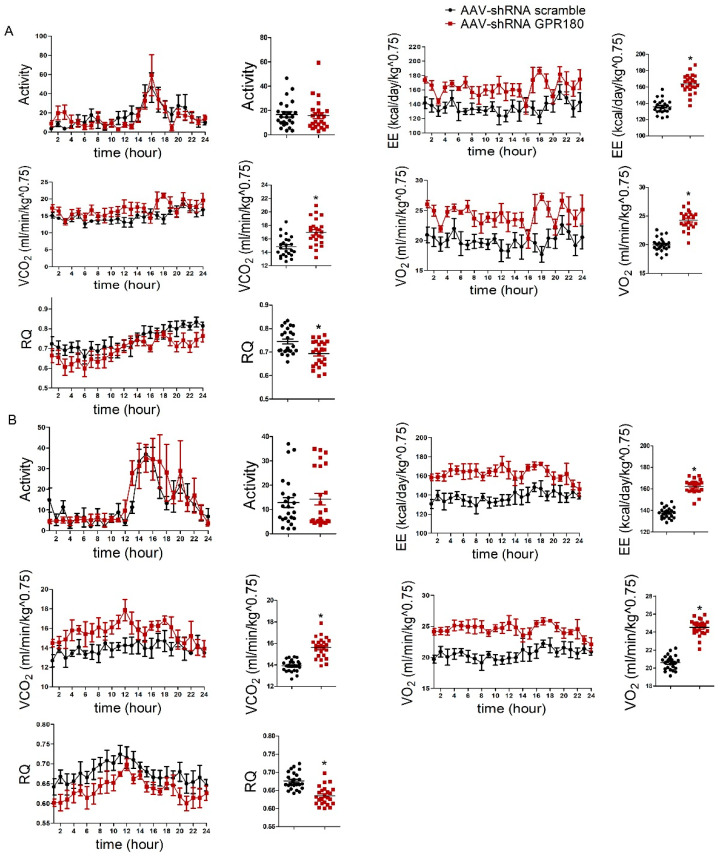
Effects of hepatic *Gpr180* knockdown on energy metabolism in mice fed NCD or HFD. Energy metabolism in hepatic *Gpr180* knockdown mice fed (**A**) NCD or (**B**) HFD mice. Results are shown as the mean ± SEM and analyzed by one-way ANOVA and Student’s unpaired *t*-test; *n* = 5 per group, * *p* < 0.05.

Real-time analysis of energy expenditure indicated that hepatic *Gpr180* deficiency significantly increased oxygen consumption, carbon dioxide production, and energy expenditure, while decreasing respiratory quotients in mice fed either NCD (Figure 3A) or HFD (Figure 3B). No obvious changes in animal activity were observed (Figure 3A,B). These results indicate that hepatic *Gpr180* deficiency enhances energy expenditure in mice fed either NCD or HFD.

### 3.4. Hepatic Gpr180 Deficiency Reduces Body Fat Mass

**Figure 4:**
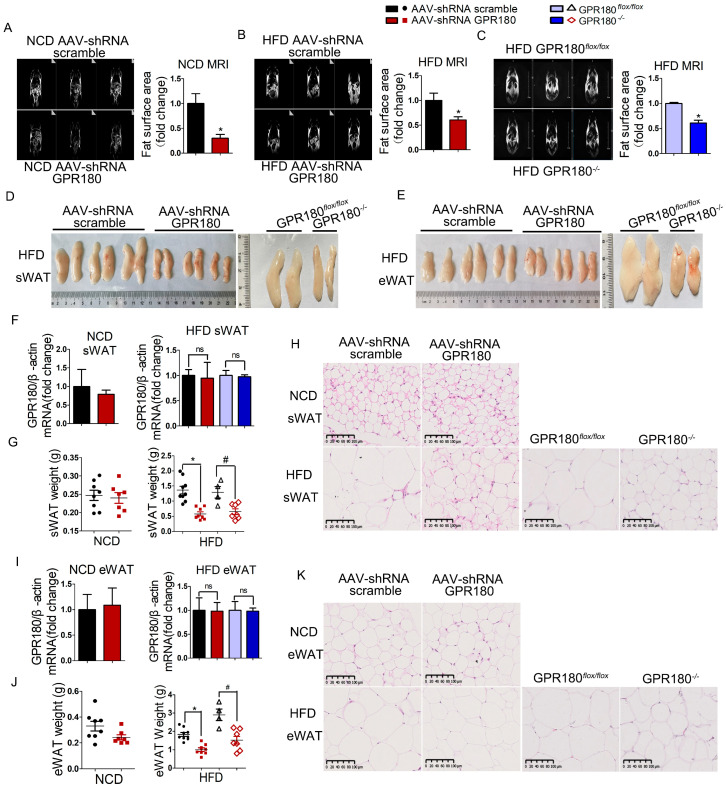
Reduction of body fat by *Gpr180* knockdown in mice. Fat distribution in mice (*n* = 3) fed (**A**) NCD or (**B**,**C**) HFD. (**D**) Sizes of sWAT and (**E**) eWAT tissues in mice fed HFD. (**F**,**I**) *GPR180* mRNA expression (*n* = 4–6), (**G**,**J**) tissue weight (*n* = 4–9), and (**H**) adipocyte size in sWAT and (**K**) eWAT. Results are shown as the mean ± SEM and analyzed by one-way ANOVA and Student’s unpaired *t*-test, * and **^#^** *p* < 0.05; ns: not significant.

The fat area measured by the MRI in *Gpr180*-deficient mice fed NCD was reduced by 70.1 ± 21.4% compared with the scramble control (Figure 4A), indicating a marked reduction of fat mass in lean mice. Meanwhile, HFD-induced obese mice exhibited abundant fat distribution, as evidenced by larger and brighter white areas in the MRI scans. In mice fed HFD, knockdown of hepatic *Gpr180* by shRNA reduced the fat mass by 39.5 ± 15.9% relevant to the scramble control (Figure 4B). A similar reduction in fat mass was observed in *Gpr180*^−/−^ mice fed HFD (Figure 4C). Furthermore, the sizes and weights of subcutaneous and epididymal white adipose tissues (sWAT and eWAT, respectively) were notably reduced in *Gpr180*-deficient mice fed HFD compared with control mice (Figure 4D,E,G,J). As demonstrated by hematoxylin–eosin (HE) staining, the size of adipocytes within subcutaneous and visceral fat tissues was significantly reduced in AAV9-shRNA *Gpr180*-treated mice and *Gpr180*^−/−^ mice fed HFD compared with the control group (Figure 4H,K). These effects were not detected in lean mice fed NCD (Figure 4G,J,H,K).

Expression levels of *Gpr180* mRNA in the adipose tissues of mice fed either NCD or HFD remained unaltered (Figure 4F,I). This observation suggests that reduction of adipose tissue mass occurs indirectly via hepatic *Gpr180* deficiency rather than its direct action in adipose tissues. Our results thus suggest that knockdown of hepatic *Gpr180* reduces the subcutaneous and visceral fat mass in obese mice.

### 3.5. Hepatic Gpr180 Deficiency Reduces Plasma and Liver Lipid Contents in HFD-Induced Obese Mice

**Figure 5:**
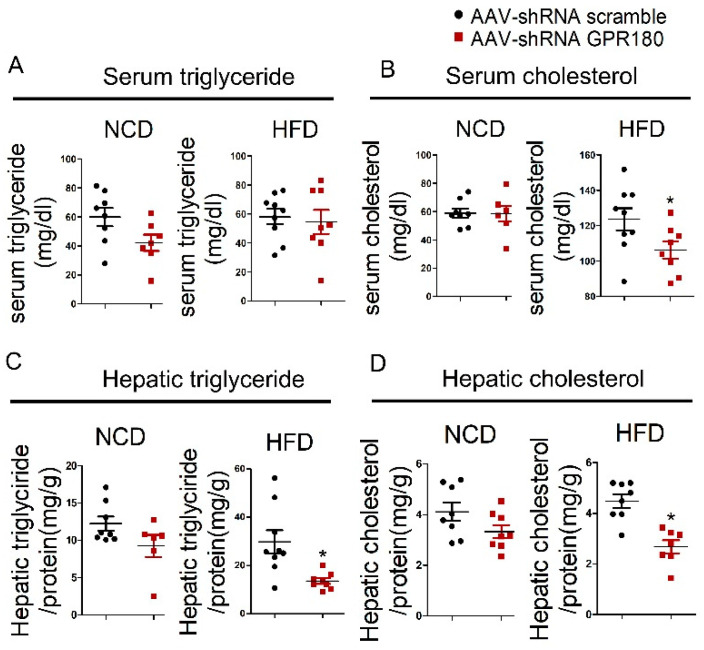
Plasma and liver lipid contents. (**A**) Plasma triglyceride, (**B**) cholesterol, (**C**) liver triglyceride, and (**D**) cholesterol contents after 12.5 weeks of treament with AAV9-*shGpr180* or control scramble shRNA in mice fed NCD or HFD. Results are shown as the mean ± SEM and analyzed by one-way ANOVA and Student’s unpaired *t*-test; *n* = 6–9, * *p* < 0.05.

To assess the effect of hepatic *Gpr180* knockdown on lipid homeostasis, we measured triglyceride and cholesterol concentrations in the plasma and liver tissues. Knockdown of *Gpr180* in the liver did not significantly affect plasma triglyceride contents in mice fed either NCD or HFD (Figure 5A). However, deficiency of hepatic *Gpr180* significantly reduced circulating and hepatic cholesterol contents in mice fed HFD, while demonstrating no effect in mice fed NCD (Figure 5B,D). Hepatic triglyceride levels were also reduced following knockdown of hepatic *Gpr180* in mice fed HFD (Figure 5C). These results indicate that hepatic *Gpr180* deficiency reduces cholesterol levels in the circulation and liver of HFD-induced obese mice, as well as the liver triglyceride contents.

**Figure 6:**
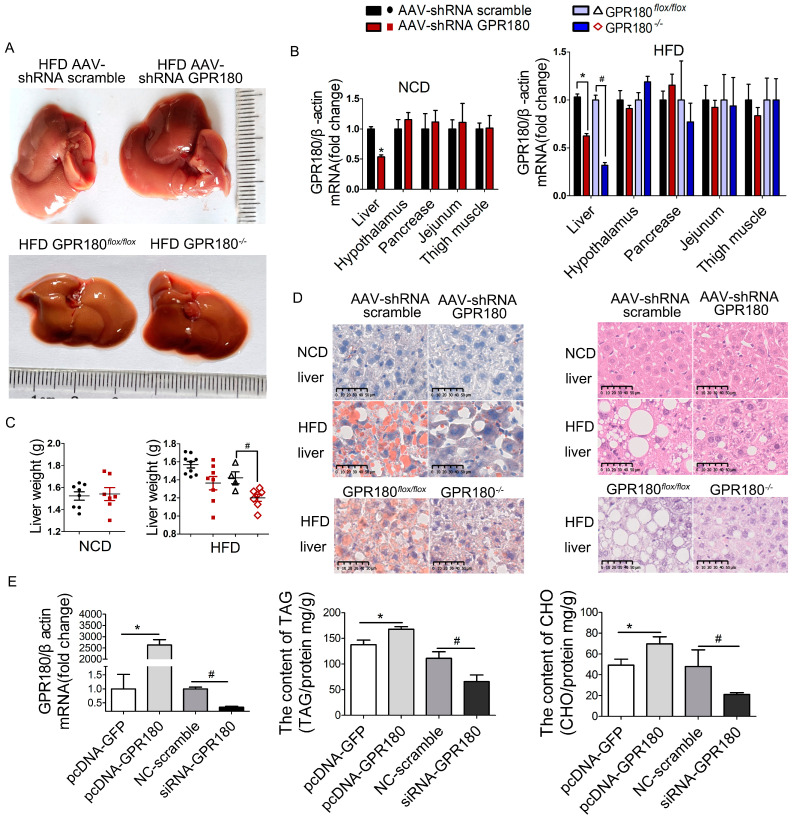
Amelioration of hepatic lipid deposition by *Gpr180* knockdown. (**A**) Liver appearance in mice fed HFD. (**B**) *Gpr180* knockdown efficiency in different tissues (*n* = 3–6). (**C**) Liver weight in mice fed NCD or HFD mice (*n* = 4–9). (**D**) Liver sections stained with oil red O and H&E. (**E**) Hepa1-6 cells were transfected with a *Gpr180* overexpression plasmid or *Gpr180*-specific siRNA for 24 h, then treated with oleic and palmitic acids for 24 h before harvesting for analysis of transfection efficiency and triglyceride and cholesterol contents (*n* = 5 for each condition). Results are shown as the mean ± SEM and analyzed by one-way ANOVA and Student’s unpaired *t*-test; * and **^#^** *p* < 0.05. TAG, triglyceride; CHO, cholesterol.

Morphologically, dense opalescent lipid droplets appeared on the liver surface of obese mice fed HFD. This effect was not observed in animals treated with AAV9-shRNA *Gpr180* or in *Gpr180*^−/−^ mice (Figure 6A), indicating an amelioration of liver steatosis. To determine whether changes in the metabolic phenotype were mediated by hepatic *Gpr180* deficiency, we examined the efficiency of *Gpr180* knockdown in the mouse liver by RT-PCR. *Gpr180*-specific shRNA markedly decreased the expression of hepatic *Gpr180* mRNA by 46.3 ± 4.8% and 38.9 ± 4.5% in mice fed NCD or HFD, respectively (Figure 6B). Compared with the *Gpr180^flox/flox^* group, *Gpr180* mRNA levels in the livers of *Gpr180*^−/−^ mice fed HFD decreased by 68.2 ± 5.4%. No significant change in *Gpr180* mRNA levels was detected in other tissues.

In *Gpr180*^−/−^ mice fed HFD, liver weight was significantly reduced. However, this effect was not significant among AAV9-*Gpr180 shRNA*-treated mice, whether fed NCD or HFD (Figure 6C). Oil red O staining further revealed abundant large lipid droplets in the livers of mice fed HFD but not those fed NCD. Moreover, the amount of lipid droplets in the livers of HFD mice was significantly reduced after treatment with *Gpr180* shRNA (Figure 6D). Hepatocyte-specific deficiency of *Gpr180* also substantially reduced lipid contents in the livers (Figure 6D). H&E staining did not reveal vacuolization caused by lipid droplets in the livers of NCD mice. In contrast, HFD mice demonstrated considerable vacuolization in hepatic tissue, as evidenced by a large number of vacuoles and extended vacuole surface area. This HFD-induced vacuolization was markedly reduced by hepatic *Gpr180* deficiency (Figure 6D).

The direct action of GPR180 in lipid metabolism was further examined using the mouse hepatocyte cell line, Hepa1-6. Transfection with *Gpr180*-pcDNA or *Gpr180*-specific siRNA led to efficient upregulation or downregulation of *Gpr180* mRNA (Figure 6E). The altered levels of *Gpr180* mRNA induced subsequent increases or decreases in intracellular triglyceride and cholesterol levels, respectively (Figure 6E).

To determine whether hepatic steatosis in mice progressed to steatohepatitis, we performed immunohistochemical staining of F4/80, the molecular marker for macrophages. F4/80-positive cells were not detected in the hepatic tissues of HFD mice, with or without *Gpr180* deficiency, indicating an absence of obvious inflammation (Supplementary Figure S2). Moreover, Sirius red and Masson’s trichrome staining did not reveal obvious collagen deposition in the livers of these animals (Supplementary Figure S3), suggesting the absence of liver fibrosis.

### 3.6. GPR180 Promotes Lipid Biosynthesis in Hepatocytes

**Figure 7:**
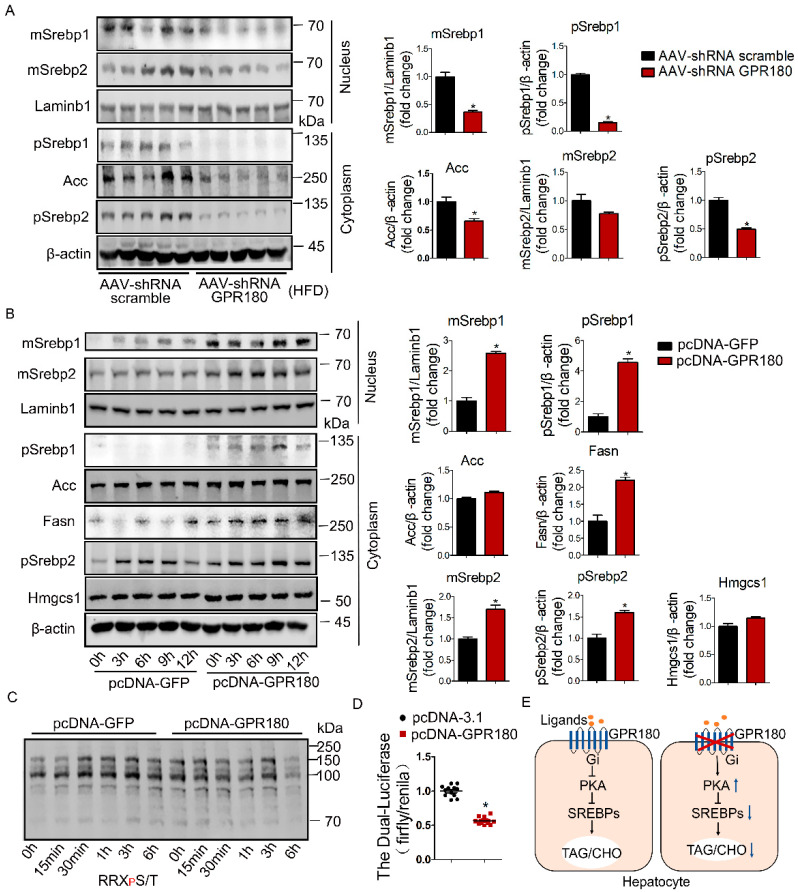
Induction of lipid biosynthesis by *Gpr180* overexpression in hepatocytes. (**A**) Protein expression of SREBP1, SREBP2, and ACC in the livers of *Gpr180*-deficient and control mice fed HFD (*n* = 5). (**B**) Hepa1-6 cells were transfected with the *Gpr180* overexpression plasmid for 30 h, cultured in serum-free medium for 12 h and then in 10% serum-containing medium, and analyzed for lipid-synthesis-related proteins at different time points by Western blotting (*n* = 5). (**C**) PKA activity in Hepa1-6 cells overexpressing *Gpr180* was evaluated by immunoblot of PKA substrate antibody at different time points. (**D**) Luciferase activity was evaluated in HEK293T cells overexpressing *Gpr180* (*n* = 12 for each condition). Data are shown as the mean ± SEM and analyzed by one-way ANOVA and Student’s unpaired *t*-test; * *p* < 0.05 (**A**,**B**,**D**). (**E**) Schematic presentation of study results. *Gpr180* knockdown in the liver ameliorates HFD-induced obesity and lipid accumulation through the Gi-PKA-SREBP pathway. Precursor SREBP1, pSREBP1; mature SREBP1, mSREBP1; Precursor SREBP2, pSREBP2; mature SREBP2, mSREBP2.

To determine whether the improvement in hepatic lipid deposition was mediated by the SREBP signaling pathway, we analyzed the cytoplasmic and nuclear abundance of related proteins in the livers of mice fed HFD. Knockdown of hepatic *Gpr180* caused a marked decrease in the abundance of SREBP proteins, including precursor and mature SREBP1 (pSREBP1, mSREBP1), pSREBP2, and mSREBP2 (Figure 7A). This effect was associated with a subsequent reduction in its target gene acetyl-CoA carboxylase (*Acc*), a key enzyme in fatty acid biosynthesis (Figure 7A).

In vitro, Hepa1-6 cells overexpressing *Gpr180* significantly increased cytoplasmic and nuclear SREBP1 and SREBP2 (pSREBP1, mSREBP1, pSREBP2, and mSREBP2). Furthermore, ACC, fatty acid synthase (FASN), and 3-hydroxy-3-methylglutaryl-CoA synthase (HMGCS1)—enzymes involved in lipid biosynthesis—were also upregulated to a certain extent (Figure 7B). These results suggest that *Gpr180* promotes the production of triglycerides and cholesterol by activating the SREBP pathway in hepatocytes.

Previously, it was reported that overexpression of GPR180 in HEK293T cells caused a decrease in cAMP abundance following treatment with 1 mM/L and 10 mM/L of L-lactic acid, suggesting that GPR180 may be a Gi-type GPCR [18]. Therefore, we next examined the effect of *Gpr180* overexpression on PKA activity by measuring the phosphorylation levels of PKA substrates under the presence of serum. In the presence of serum stimulation, overexpression significantly reduced the phosphorylation levels of PKA substrates at the 6 h time point (Figure 7C). Further, overexpression of *Gpr180* significantly inhibited CREB-driven promoter luciferase activity (Figure 7D). These data indicate that GPR180 may be a Gi-coupled receptor.

## 4. Discussion

The results of this study demonstrate that hepatic GPR180 contributes to the control of lipid metabolism. Using two approaches to suppress *Gpr180*: AAV9-shRNA *Gpr180* and *albumin-Gpr180*^−/−^, we showed that hepatic *Gpr180* deficiency renders mice resistant to HFD-induced liver steatosis, which is associated with metabolic benefits including enhanced insulin sensitivity and energy metabolism, as well as reduced adiposity. Our findings thus extend the physiological functions of orphan receptor GPR180 from vascular homeostasis to the control of lipid metabolism. By stimulating lipid biosynthesis in the liver, GPR180 contributes to the control of global lipid homeostasis. Consistently, recent GWAS studies have indicated that SNPs in *GPR180* may be associated with dyslipidemia. For instance, a meta-analysis of 69,414 East Asian individuals in 24 GWASs has revealed that *GPR180* rs12341267 is associated with blood triglyceride level [19]. Another study found that *GPR180* rs2298058 and rs9561643 could be related to the transcriptional activation of *GPR180* and elevation of serum triglyceride levels [14]. In addition, the study by Balazova et al. revealed that GPR180 in adipocytes can promote the development of beige fat cells, leading to incremental heat production and improved energy metabolism [20]. Collectively, these observations suggest that GPR180 is critical for lipid homeostasis, although its physiological function varies in distinct tissues. Notably, the reduction in adiposity and the observed metabolic advantages appear to be closely linked to the improved hepatic lipid metabolism resulting from *Gpr180* deficiency in the liver. Moreover, the reduction in adiposity associated with hepatic GPR180 deficiency in our study further supports a metabolic benefit via improved hepatic lipid metabolism. While our findings highlight the critical role of hepatic metabolism in driving these beneficial changes, the mechanism by which hepatic GPR180 deficiency reduces fat mass remains unknown. In addition to metabolic regulation through triglycerides and fatty acids, the liver also controls the body’s metabolism through endocrine signaling. For example, Fibroblast Growth Factor 21 (FGF21) is a peptide hormone synthesized by various organs, including the liver, with the ability to regulate energy homeostasis [21]. Angiopoietin-like protein 4 (ANGPTL4) is a secreted protein synthesized in the liver that can inhibit lipoprotein lipase and activate cAMP-stimulated lipolysis in adipocytes [22]. Future investigation should focus on the identification of the key secretory hepatokines altered by GPR180, which will provide insights into how hepatic GPR180 regulates the organism metabolism and energy balance.

Although the intracellular signaling pathways activated by GPR180 remain unclear, our study indicates that GPR180 may activate the SREBP1 and SREBP2 in hepatocytes. Both in vitro and in vivo experiments demonstrated that GPR180 deficiency in hepatocytes significantly decreased the nuclear levels of SREBP1 and SREBP2, leading to substantial reduction in their downstream targets ACC. The second messenger that mediates the effect of GPR180 activation on SREBPS is currently unknown. Our observations showed that overexpression of *Gpr180* significantly reduced PKA-mediated protein phosphorylation in hepatocytes. In addition, overexpression of *Gpr180* suppressed the CREB activity, the downstream target of PKA. Together with previous observations demonstrating that PKA-mediated activity inhibits the expression of SREBPs [17,23,24,25], we thus propose that GPR180 may activate SREBPs via Gi-PKA signaling to regulate the lipid biogenesis in hepatocytes. A group of GPCRs acts as receptors for gut peptides, bile acids, chemokines, free fatty acids, and intermediate metabolites, regulating hepatic lipid metabolism and development of NAFLD. For example, Free Fatty Acid Receptor 2 (FFAR2), one of the main receptors for short-chain fatty acids, is coupled with the Gi protein [26]. Activation of FFAR2 can inhibit the cAMP-PKA signaling pathway and its downstream signaling cascades. These findings support the concept that various metabolites and relevant GPCRs function to alter Gi-PKA-SREBP signaling pathways, leading to alteration in lipid metabolism and the progression of NAFLD.

Limitations exist for our study. Mice were fed with HFD to induce NAFLD. Liver steatosis was detected in these animals. However, inflammation and fibrosis were not obvious. In future experiments, we will consider extending the duration of HFD feeding and using other models, such as methionine-choline-deficient (MCD) and HFD-high-cholesterol (HC) diet, to further investigate the effect of GPR180 in inflammation and fibrosis in NAFLD.

In conclusion, our study demonstrates that *Gpr180* knockdown in the mouse liver can significantly inhibit HFD-induced obesity and liver steatosis. Mechanistically, GPR180 stimulates biosynthesis of triglyceride and cholesterol in a manner dependent on Gi-PKA-SREBPs signaling (Figure 7E). Thus, GPR180 represents a potential drug target for the treatment of lipid disorders such as obesity and NAFLD.

## Figures and Tables

**Table 1:** Relationship between *GPR180* polymorphisms and serum triglyceride levels.

Variant and Risk Allele	Beta (Increase)	Mapped Gene	Reported Trait	Study Accession
rs1341267-A	0.0184 unit	GPR180	Triglycerides	GCST004237
rs9556404-A	0.018 unit	GPR180	Triglycerides	GCST007133
rs9556404-A	0.02 unit	GPR180	Triglycerides	GCST007142
rs2298058-T	0.0245 mg/dL unit	TGDS, GPR180	Triglycerides	GCST006613

Note: These data were obtained from GWAS Catalog database (https://www.ebi.ac.uk/gwas/home (accessed on 12 May 2022)).

## Data Availability

The data presented in this study are available on request from the corresponding author. The data are not publicly available due to privacy restrictions.

## Supplementary Materials

The following supporting information can be downloaded at: https://www.mdpi.com/article/10.3390/nu15081838/s1.
