# Supplementary material for: Hepatic G Protein-Coupled Receptor 180 Deficiency Ameliorates High Fat Diet-Induced Lipid Accumulation via the Gi-PKA-SREBP Pathway
Source: Nutrients. 2023 Apr 11;15(8):1838. doi: 10.3390/nu15081838 (PMC10144310; doi:10.3390/nu15081838)
Supplement: Supplementary file 1 [file nutrients-15-01838-s001.zip › nutrients-2304345-supplementary.pdf]

Hepatic G Protein-Coupled Receptor 180 Deficiency Ameliorates High Fat Diet-Induced Lipid Accumulation via the Gi-PKA-SREBP Pathway

Yunhua Zhang 1,2,†, Ziming Zhu 1,†, Lijun Sun 1, Wenzhen Yin 1, Yuan Liang 1, Hong Chen 1, Yanghui Bi 1, Wenbo Zhai 1, Yue Yin 3,* and Weizhen Zhang 1,*

[1] Department of Physiology and Pathophysiology, School of Basic Medical Sciences, and Key Laboratory of Molecular Cardiovascular Science, Ministry of Education, Peking University, Beijing 100191, China

[2] The Key Laboratory of Xinjiang Endemic & Ethnic Diseases and Department of Biochemistry, Shihezi University School of Medicine, Shihezi 832002, China.

[3] Department of Pharmacology, School of Basic Medical Sciences, and Key Laboratory of Molecular Cardiovascular Science, Ministry of Education, Peking University, Beijing 100191, China

***Corresponding authors**

Yue Yin

yueyin@bjmu.edu.cn

Weizhen Zhang

weizhenzhang@bjmu.edu.cn

† These authors contributed equally to this work.

Supplementary Figure S1. Glucose metabolism in mice fed NCD and HFD was evaluated.

Supplementary Figure S2. Immunohistochemical detection of F4/80 in the livers of NCD and HFD mice.

Supplementary Figure S3. Levels of fibrosis in the livers of NCD and HFD mice assessed via Sirius red and Masson's trichrome staining.

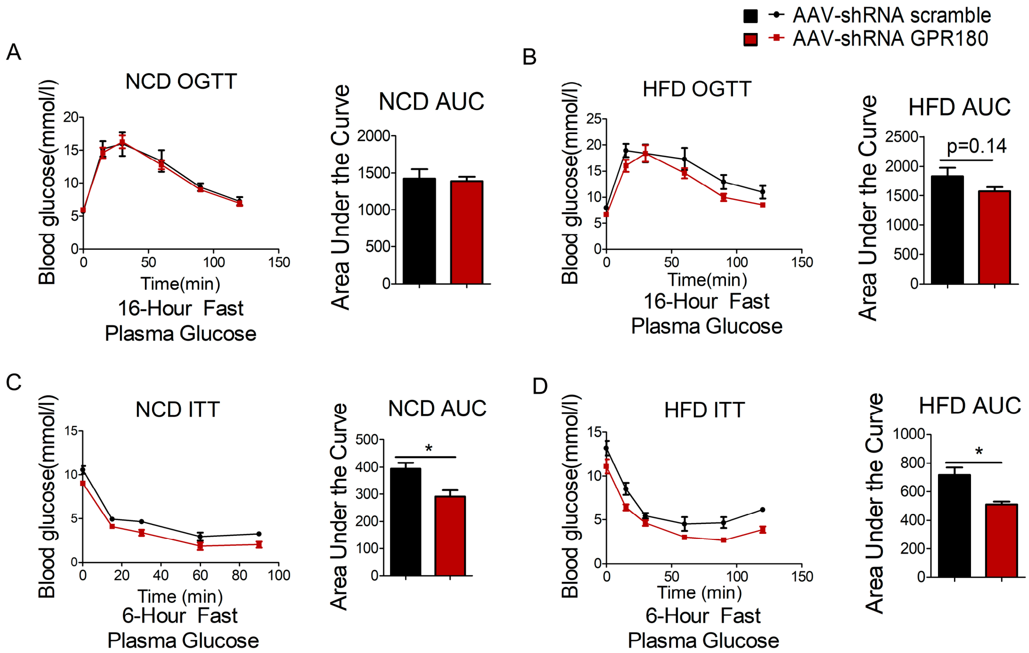

Supplementary Figure S1. Glucose metabolism in mice was evaluated. A-B: Oral glucose tolerance test (OGTT) used in NCD (A) and HFD (B) mice. C-D: Insulin tolerance test (ITT) used in NCD (C) and HFD (D) mice. Results are shown as the mean±SEM (n=5; *P<0.05). AUC, area under the curve.

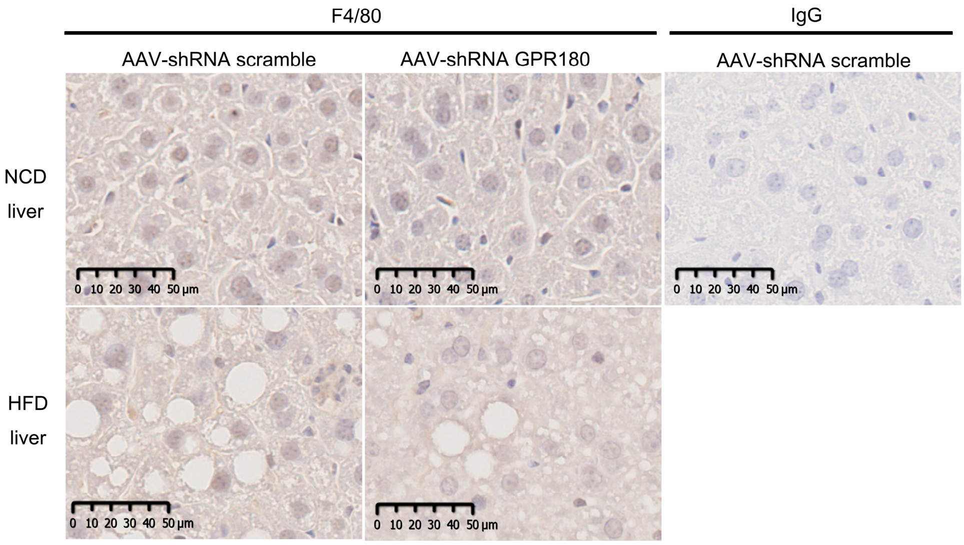

Supplementary Figure S2. Immunohistochemical detection of F4/80 in the livers of NCD and HFD mice.

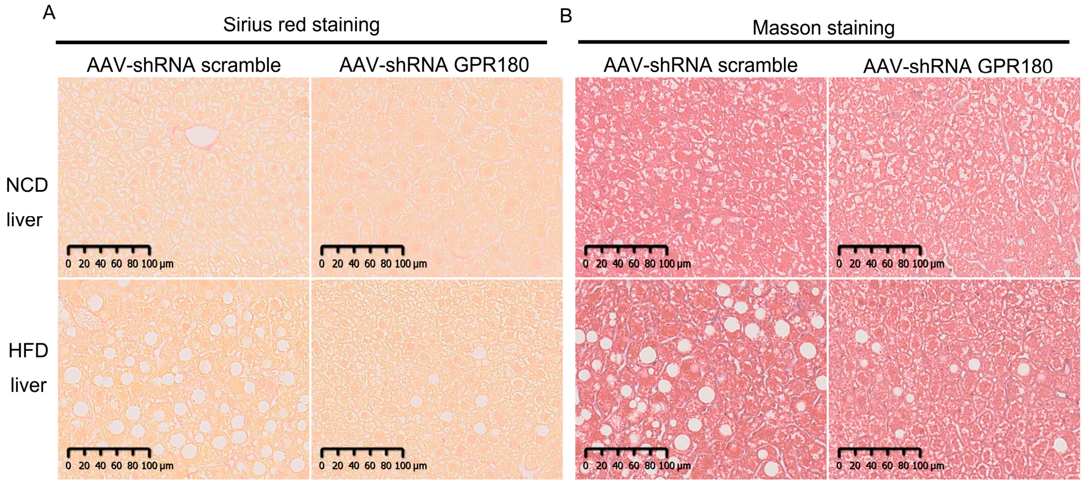

Supplementary Figure S3. Levels of fibrosis in the livers of NCD and HFD mice assessed. A-B: Sirius red (A) and Masson's trichrome staining (B) were used.
